# Mental health and substance use screening in HIV primary care before and during the early COVID-19 pandemic

**DOI:** 10.1186/s12913-023-09477-6

**Published:** 2023-05-16

**Authors:** Alexandra N. Lea, Tory M. Levine, Thibaut Davy-Mendez, Amy Leibowitz, Andrea Altschuler, Jason Flamm, C. Bradley Hare, Mitchell N. Luu, Michael J. Silverberg, Derek D. Satre

**Affiliations:** 1 Division of Research, Kaiser Permanente Northern California, Oakland, CA USA; 2 University of North Carolina at Chapel Hill School of Medicine, Chapel Hill, NC USA; 3 Sacramento Medical Center, Kaiser Permanente Northern California, Sacramento, CA USA; 4 San Francisco Medical Center, Kaiser Permanente Northern California, San Francisco, CA USA; 5 Oakland Medical Center, Kaiser Permanente Northern California, Oakland, CA USA; 6 Department of Psychiatry and Behavioral Sciences, Weill Institute for Neurosciences, University of California, San Francisco, CA USA

**Keywords:** HIV, Screening, Substance use, Mental Health, COVID-19

## Abstract

**Background:**

Mental health and substance use disorders disproportionately affect people with HIV (PWH), and may have been exacerbated during COVID-19. The Promoting Access to Care Engagement (PACE) trial was designed to assess the effectiveness of electronic screening for mental health and substance use in HIV primary care and enrolled PWH from October 2018 to July 2020. Our objective here was to compare screening rates and results for PWH before (October 2018 – February 2020) and early in the COVID-19 pandemic (March-July 2020).

**Methods:**

Adult (≥ 18 years) PWH from 3 large HIV primary care clinics in a US-based integrated healthcare system were offered electronic screening online or via in-clinic tablet computer every 6 months. Screening completion and results (for depression, suicidal ideation, anxiety, and substance use) were analyzed using logistic regression with generalized estimating equations to estimate prevalence ratios (PR) before and after the start of the regional COVID-19 shelter-in-place orders on March 17, 2020. Models adjusted for demographics (age, sex, race/ethnicity), HIV risk factors (men who have sex with men, injection drug use, heterosexual, other), medical center, and modality of screening completion (online or tablet). We conducted qualitative interviews with providers participating in the intervention to evaluate how the pandemic impacted patient care.

**Results:**

Of 8,954 eligible visits, 3,904 completed screenings (420 during COVID, 3,484 pre-COVID), with lower overall completion rates during COVID (38% vs. 44%). Patients completing screening during COVID were more likely to be White (63% vs. 55%), male (94% vs. 90%), and MSM (80% vs., 75%). Adjusted PRs comparing COVID and pre-COVID (reference) were 0.70 (95% CI), 0.92 (95% CI), and 0.54 (95% CI) for tobacco use, any substance use, and suicidal ideation, respectively. No significant differences were found by era for depression, anxiety, alcohol, or cannabis use. These results were in contrast to provider-reported impressions of increases in substance use and mental health symptoms.

**Conclusion:**

Findings suggest PWH had modest declines in screening rates early in the COVID-19 pandemic which may have been affected by the shift to telemedicine. There was no evidence that mental health problems and substance use increased for PWH in primary care.

**Trial registration:**

NCT03217058 (First registration date: 7/13/2017); https://clinicaltrials.gov/ct2/show/NCT03217058

## Introduction

Depression, anxiety, and substance use disorders disproportionately affect people with HIV (PWH) compared with people without HIV, and are associated with poor HIV clinical outcomes (e.g., HIV viral control) [1, 2] and increased mortality. [3, 4] The COVID-19 pandemic was anticipated to exacerbate these issues among PWH.

At the pandemic outset, concerns were raised about potential increases in depression, anxiety, and alcohol and drug use in the general population and in vulnerable subgroups. [5, 6] In the first year of the pandemic, global prevalence of anxiety and depression increased by 25%. [7] Use of alcohol and cannabis also increased, particularly in people with anxiety and depression. [8] For PWH, worsening of mental health care access and increases in alcohol and substance use were also anticipated. [9–12] Despite these concerns, few studies have examined the impact of the COVID-19 pandemic on mental health and substance use among PWH, nor have studies evaluated potential changes in screening for these problems. In studies of early COVID-19 responses in PWH, specifically, heightened anxiety and depression were reported but changes in in substance use were not consistently found. [13–16] To our knowledge, no studies have examined differences in screening rates and prevalence of substance use and mental health symptoms in PWH delivered in the context of primary care. The current study leveraged data from an HIV primary care-based trial implemented prior to and continuing through the start of the early COVID era in order to address these important questions.

The Promoting Access to Care Engagement (PACE) trial implemented computerized, self-reported screening for mental health disorders and substance use in conjunction with HIV primary care appointments, with referral to behavioral health specialists for treatment. [17] The PACE trial implemented screening at three HIV primary care clinics prior to the onset of the COVID-19 pandemic, which continued into the early months of the pandemic. The current analysis aimed to compare differences in screening rates and prevalence of self-reported depression, suicidal ideation, anxiety, and substance use among PWH before and early in the pandemic. We supplemented these findings with qualitative interviews with HIV primary care providers regarding their perspectives on the pandemic’s impact on the health of their patients.

## Methods

The PACE trial combined mental health and substance use screening measures into a single, self-report questionnaire systematically administered via online secure message or in-clinic tablets. The questionnaire was offered every 6 months to PWH, in connection with their regular primary care appointments. [17] PACE was completed in the three largest HIV primary care clinics (Oakland, Sacramento, and San Francisco) in Kaiser Permanente Northern California, which collectively serve over 5000 PWH (roughly half of all PWH in the health system). Eligible participants were all adult (≥ 18 years) PWH attending HIV care visits with participating providers from October 2018 to July 2020, at which time the study was scheduled to be completed. PWH could complete the questionnaire via online secure messaging up to two weeks prior to a scheduled visit or in clinic on a tablet. After the onset of the COVID-19 pandemic, telemedicine visits were included since in person visits were limited for several months.

The questionnaire combined several measures into a single instrument that was sent to the patient prior to their visit: The Tobacco, Alcohol, Prescription medication and other Substance use (TAPS) instrument [18] was used to assess substance use, the Patient Health Questionnaire (PHQ-9) [19] was used for depression and the Generalized Anxiety Disorder (GAD-2) [20] was used for anxiety. These measures have previously been validated for electronic self-administration. [21] The TAPS tool, specifically, has high levels of specificity and sensitivity across substances, as well as a high level of patient acceptability, with 99% of participants comfortable answering questions and 95% of participants comfortable sharing their results with their provider. [22] We defined a positive screen for depression as PHQ-9 score ≥ 10, suicidal ideation as PHQ-9 question 9 score > 0, anxiety as GAD-2 score ≥ 3, and substance use as a TAPS score ≥ 1 for each substance. “Any substance use” was defined as a positive screen for at least one substance. To evaluate how representative the screened sample was of the eligible primary care population of PWH at the three study sites, we compared demographic and clinical characteristics of those who completed the questionnaire with those who did not complete it, using chi-square and t-tests for comparison between groups.

We compared the proportion of eligible visits with a completed screen, and the proportion of positive screens for depression, suicidal ideation, anxiety, and substance use before and after the start of regional COVID-19 shelter-in-place orders on March 17, 2020. We estimated adjusted prevalence ratios (aPRs) using generalized estimating equations (GEE) with robust standard errors to account for multiple visits contributed by patients. Models were adjusted for demographics (age, sex, self-reported race/ethnicity); HIV risk factors (men who have sex with men [MSM], injection drug use [IDU], Heterosexual, Other); Medical Center (Oakland, Sacramento, San Francisco); and screening modality (online, tablet). Analyses were performed using Proc Genmod in SAS v. 9.4 (Cary, NC).

To evaluate how the COVID-19 pandemic impacted patient care, particularly screening and treatment for mental health and substance use, we also conducted interviews with an HIV care provider from each site (including authors JF and MNL). We used convenience sampling to select a participating HIV provider at each study site after the onset of the pandemic. Verbal informed consent was obtained from providers, who did not receive compensation for participation. Interviews lasted approximately 30 min and were audio recorded and transcribed.

We used a thematic approach to analyze qualitative interview data, combining deductive and inductive reasoning, for coding and analysis. [23, 24] This approach was chosen to identify and evaluate both explicit and implicit perspectives provided by interviewees, which were identified as both themes and sub-themes. Two authors (AA & ANL) independently coded all of the transcripts. Differences in coding were resolved via consensus, and final codebooks were established. Data analysis was managed using NVivo statistical software version 12 (QSR International) and followed standard methods for qualitative research to ensure analysis was systematic and verifiable. [25, 26]

All study procedures were approved by the Kaiser Permanente Northern California and University of California, San Francisco Institutional Review Boards and carried out in accordance with required guidelines and regulations.

## Results

Over the course of the entire intervention period (October 2018 through July 2020), there were 8,954 eligible visits (7,849 pre-COVID and 1,105 during early COVID) scheduled by 4,134 unique PWH. Of the 7,849 eligible visits pre-COVID, 3,484 screenings were completed (44%) and of the 1,105 eligible visits during early COVID, 420 screenings were completed (38%).

### Demographics

Regarding the representativeness of individuals who completed a screen compared with the eligible primary care population of PWH, individuals who did not complete a screen were less likely to be White (45% vs. 56%) and more likely to be Hispanic (22% vs. 15%) compared with PWH who had at least one screen (p < 0.01) (Table 1). Minor differences were also noted in age and HIV control (p < 0.01), while gender and HIV risk group did not significantly differ between PWH who did or did not complete a screen. Among PWH who completed a screen, the pre- and early-COVID samples had some similarities in demographics (age [median = 55 vs. 58], and risk group [6% vs. 5% IDU] (p  ≤ 0.01); however, patients during COVID were more likely to be White (63% vs. 55%), male (94% vs. 90%), and MSM (80% vs. 75%) than pre-COVID (p ≤ 0.01) (Table 2).

**Table 1:** Demographic and clinical characteristics of persons with HIV eligible for primary care-based substance use and mental health screening, stratified by screening completion (N = 4134)

Characteristic	Completed ≥ 1 screen N = 2865	No screen completed N = 1269	p-value
Men	2629 (92%)	1152 (91%)	0.2971
Race and ethnicity			< .0001
Asian or Pacific Islander	198 (7%)	88 (7%)	
Black	558 (19%)	278 (22%)	
Hispanic	419 (15%)	277 (22%)	
White	1602 (56%)	574 (45%)	
Other/unknown	88 (3%)	52 (4%)	
Age, years	54 (13)	51 (13)	< .0001
HIV risk group			0.0615
MSM	2170 (76%)	922 (73%)	
IDU	185 (6%)	103 (8%)	
Heterosexual or other	510 (18%)	244 (19%)	
CD4 count, ^a^ cells/µL	674 (306)	639 (302)	0.0023
HIV RNA < 200 copies/mL ^a^	2678 (96%)	1111 (93%)	0.0001
Insurance type			0.0405
Private	1968 (69%)	885 (70%)	
Medicare	732 (26%)	287 (23%)	
Medicaid	142 (5%)	85 (7%)	
Other	23 (1%)	12 (1%)	
NDI quartile ^b^			0.9927
1 (least deprived)	717 (25%)	315 (25%)	
2	726 (25%)	319 (25%)	
3	704 (25%)	317 (25%)	
4 (most deprived)	712 (25%)	317 (25%)	

Numbers are N (%) or mean (SD). Abbreviations: IDU, injection drug use; MSM, men who have sex with men; NDI, neighborhood deprivation index; SD, standard deviation.

^a^ Closest measurement within six months before or after screening date, or first eligible visit for patients with no completed screen.

^b^ Calculated according to Messer et al. (2006) and divided in quartiles based on the distribution of the entire patient sample.

**Table 2:** Characteristics of patients screened before COVID and during early COVID, including multiple screens per patient.

	Before COVID		Early COVID	
Eligible visits, n	7849	-	1105	-
Screenings completed, n (%)	3484	44%	420	38%
**Patient Characteristics, n (%)**				
*Age, median (IQR)*	55	46–62	58	48–66
*Sex*				
Male	3147	90%	395	94%
Female	337	10%	25	6%
*Risk group*				
MSM	2621	75%	338	80%
Heterosexual	465	13%	34	8%
IDU	217	6%	21	5%
Other	181	5%	27	6%
*Race*				
White	1914	55%	264	63%
Black	718	21%	57	14%
Hispanic	513	15%	66	16%
Other	181	5%	27	6%
*Appointment Facility*				
Oakland	1516	44%	152	36%
Sacramento	878	25%	66	16%
San Francisco	1090	31%	202	48%
*Answer Modality*				
Exam Room Computer	19	1%	0	0%
Online	2265	65%	419	100%
Tablet	1200	34%	1	0%

Notes: IQR, interquartile range; MSM, men who have sex with men; IDU, intravenous drug use

### Screening rates

Of 7,849 eligible visits pre-COVID, 3,484 (44%) had a completed screen, compared with 420 of 1,105 eligible visits (38%) during early COVID (Table 2). By clinic, screening rates pre- and early-COVID decreased in Oakland (41% of all eligible appointments vs. 30%) and Sacramento (59% vs. 39%) but increased in San Francisco (41% vs. 48%) (Table 3). Overall screening completion decreased during early COVID vs. pre-COVID (38% vs. 44% of eligible visits; adjusted prevalence ratio [aPR] = 0.74, 95% CI = 0.65–0.85).

**Table 3:** Screening completion rates before COVID and during early COVID by site

	Before COVID			Early COVID		
Site	Attached	Completed.	Rate	Attached	Completed	Rate
*OAK*	3,662	1,516	41%	513	152	30%
*SAC*	1,497	878	59%	169	66	39%
*SFO*	2,690	1,090	41%	423	202	48%
**Total**	**7,849**	**3,484**	**44%**	**1,105**	**420**	**38%**

Notes: *All p-values < .0001

### Screening results

**Fig. 1:**
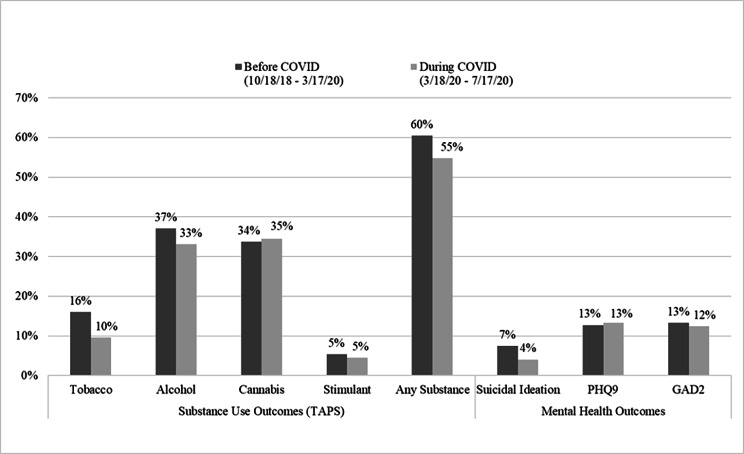
Unadjusted Screening Results Before COVID vs. During Early COVID

No significant differences were found during early COVID vs. pre-COVID in prevalence of depression (13% vs. 13%), anxiety (12% vs. 13%), alcohol use (33% vs. 37%) or cannabis use (35% vs. 34%) (Fig. 1). During early COVID, patients were less likely to report tobacco use (aPR = 0.70, 95% CI = 0.53–0.93), any substance use (aPR = 0.92, 95% CI = 0.84–1.00), and suicidal ideation (aPR = 0.54, 95% CI = 0.33–0.86) (Table 4).

**Table 4:** Prevalence Ratios for Substance Use and Depression/Anxiety during early COVID compared to before COVID

	Unadjusted PR (95% CI)	p	Adjusted PR (95% CI)^a^	p	
**Substance Use (TAPS ≥ 1)**					
Tobacco	**0.60 (0.45–0.80)**	**0.001**	**0.70 (0.53–0.93)**	**0.013**	
Alcohol	0.89 (0.78–1.02)	0.105	0.92 (0.81–1.05)	0.222	
Cannabis	1.09 (0.90–1.17)	0.712	1.03 (0.90–1.17)	0.674	
Stimulant	0.84 (0.53–1.32)	0.443	0.78 (0.50–1.22)	0.271	
Any Substance ^b^	**0.91 (0.83–0.99)**	**0.026**	**0.92 (0.84–1.00)**	**0.043**	
**Suicidal Ideation, Depression, Anxiety**					
Suicidal Ideation	**0.54 (0.34–0.87)**	**0.012**	**0.54 (0.33–0.86)**	**0.01**	
Depression	1.05 (0.82–1.35)	0.709	1.11 (0.86–1.43)	0.413	
Anxiety	0.94 (0.72–1.22)	0.621	1.05 (0.80–1.36)	0.738	

^a^ Adjusted for age, sex, race/ethnicity, risk (MSM, IV Drug Use, Heterosexual, Other), and medical center

^b^ Any substance includes: tobacco, alcohol, cannabis, stimulants, heroin, opioids, sedatives, Rx stimulant, IV drug use, and other drug use

We identified four major themes related to the impact of COVID on the care provision from the provider interviews, with subthemes identified for each (Table 5), Providers completing qualitative interviews at all three sites identified the shift to telemedicine as a significant contributor to differences pre-COVID and during early COVID, reporting changes to the patient rooming process during the shift to virtual visits, such as the transition to virtual exam rooms as well as limited appointment times requiring medical assistants to triage non-COVID patient screening as major factors. Additionally, the option to complete the survey via in-clinic tablet was no longer available due to fears related to COVID transmission, making access to technology an important consideration. Finally, under the theme of “Impact on substance use and mental health symptoms”, increases in patient-reported mental health symptoms were also reported by all providers. Providers at Oakland and San Francisco described an increase in patients’ alcohol use and smoking, while the providers at the Sacramento site felt that there were no major shifts in alcohol or drug use reported by patients.

**Table 5:** Major themes and sub-themes identified in qualitative interviews with participating providers

Major Themes	Sub-themes
1. Access to HIV Care	Frequency of primary care visits
	Frequency of HIV labs
	Access to antiretroviral therapy
2. Shift to Telemedicine	Physician workload
	Appointment time limitations
	Ability to view screening results in EHR
	Mode of administration/Access to technology
	Staffing changes
3. Impact on Substance Use & Mental Health symptoms	Alcohol Use
	Drug use
	Depression
	Anxiety
4. Impact on Substance Use & Mental Health treatment	Impact on provider referrals to specialty care
	Impact on behavioral health services

Notes: EHR, electronic health record

## Discussion

In this study of PWH asked to complete substance use and mental health screening prior to regular HIV primary care appointments both before and after the onset of the COVID pandemic, overall mental health and substance use screening rates decreased. Self-reported suicidal ideation, tobacco use, and any substance use also decreased.

One possible explanation for the decrease in screening rates is that the questionnaire was intended to be given prior to regularly scheduled primary care visits, many of which were cancelled or delayed due to COVID transmission risk, resulting in reduced care engagement by PWH. This may have resulted in lower completion rates for appointments that shifted from in-person visits to telemedicine. Similarly, other studies have also shown decreases in care during this time for the general population [27], as well as PWH [28, 29].

Online completions required patients to have access to KP.org secure messaging to complete the questionnaire online prior to their appointment. Prior to COVID, the Oakland and Sacramento sites had similar rates for tablet (in-person) vs. online screening completion, while San Francisco skewed heavily towards online completions. During early COVID, the majority of completed appointments across all three sites shifted to a telemedicine (online) model. San Francisco was the only site that saw an increase in screening rates, which may indicate that their population was more comfortable with virtual care delivery, as compared with the other two sites.

Across all three sites, PWH who completed the study questionnaire during early COVID were more likely to be White, male, and MSM as compared with pre-COVID. It is worth noting that the demographics of the San Francisco site reflect the older, White MSM who make up the population of PWH in the city, as compared with the Oakland and Sacramento sites, who are younger and include greater proportions of racial/ethnic minority PWH, women, and heterosexual PWH [17]. Previous studies have shown PWH who do not identify as White to be less likely to utilize telehealth services compared with White patients. [28, 30] Our results also are consistent with prior findings that older PWH are more likely to attend telemedicine visits compared with younger patients due to a variety of potential factors such as familiarity with telephone appointments or lowering of barriers such as time or travel [15, 31], which may be particularly relevant in the context of COVID exposure fears.

No differences were found in self-reported levels of depression and anxiety. These results are in contrast to previous work showing increases in depression and anxiety in PWH during the early pandemic [14], including a recent meta-analysis by Lee et al. that found that the pooled prevalence rates of depression and anxiety among PWH were 16.9% (95% confidence interval [CI]: 3.8-30.0%) and 23.0% (95% CI: 12.0-34.0%), respectively [32]. However, similar results have been reported by Pizzirusso and colleagues and may be reflective of PWH being more engaged with care at the onset of the pandemic, as well as greater levels of psychological resiliency in older populations [33].

Although no differences were found in reported depression and anxiety, we did find a decrease in potential suicidal ideation during early vs. pre-COVID. Little has been published on suicidality in PWH during the early pandemic. One mixed methods study of PWH in the United Kingdom (UK) during a similar time period (May-July 2020) found 19.8% reporting suicidal thoughts at any point. [34] This study adds to this literature by examining a sample of PWH engaged in care in the United States (US) as well as being able to compare self-reported mental health outcomes both before and during the early COVID-19 pandemic. Studies in both the UK and the US have found that PWH reported higher levels of resilience and that prior experience with an infectious disease with potentially deadly consequences may have better equipped them emotionally for the pandemic compared with people without HIV. [34–36]

Finally, we also noted decreases in tobacco use and any substance use, and no differences for alcohol or cannabis use. These findings are consistent with prior work showing that smoking and illicit drug use were lower in PWH during the pandemic. [15, 16, 35] The pandemic decreased availability of illicit substances through supply chain disruption as well as lockdown and social distancing requirements limiting opportunities to engage in social use [5], factors that could have contributed to the reductions we found in this sample.

Although reasons for the contrasting observations of clinicians and patient-reported symptoms are not clear, it is possible that concerns about patient well-being during the pandemic may have influenced provider’s perceptions, or that contact with a small number of higher-severity patients (including perhaps ones who did not complete screening) influenced their perceptions.

### Strengths & limitations

The PACE trial occurred during normal primary care visits in an integrated healthcare system with a diverse patient population. We had a large sample size and a substantial number of respondents who completed screening both pre- and during early COVID. However, there were some study limitations. First, our screening process was tied to upcoming attendance at primary care visits. While uncoupling screenings from visits might have increased the pool of individuals invited to complete screening questionnaires, it could also reduce the screening completion rate because most members of the healthcare system have been accustomed to receiving screening questionnaires prior to upcoming appointments of all types through the patient portal system. Linking screenings to upcoming visits may increase motivation to complete questionnaires, since patients anticipate that providers may expect a response. Primary care visits are a key component of routine health care in most settings and having screenings tied to upcoming primary care visits could make our results more generalizable to a variety of healthcare settings.

Our results were also based on self-report, which may result in underestimation of substance use. [37] However, the same technology and approach was utilized both pre- and during-COVID, which likely resulted in similar levels of underreporting in both time periods. There is a theoretical potential for changes in reporting patterns during the pandemic, e.g., due to either increased stigmatization [38] or normalization of substance use and mental health problems [39], although this has not been reported on to date. Self-administered questionnaires may also minimize response bias and promote accurate reporting. Additionally, although findings were adjusted for demographics, HIV risk factors, setting, and screening modality, some groups were small, particularly during the COVID period, which may have led to residual confounding. Finally, the COVID study period was relatively short (four months) and therefore only reflects the early phase of the pandemic. During this time, all primary care in the region quickly shifted to a telemedicine model and guidelines and recommendations for care engagement changed rapidly. Therefore, results may also reflect a shift in the patient population to those who were more adept at accessing telehealth or who were less likely to use substances than those who did not access care during the pandemic. However, it is important to note that this screening was embedded in routine HIV primary care, which may make it more generalizable to other HIV-positive populations than a randomized clinical trial, and that PWH in our study setting had high levels of secure messaging access and patient portal use prior to COVID.

## Conclusion

We observed a decrease in substance use and mental health screening rates for PWH during early COVID, which may reflect reduced patient engagement with telemedicine in certain populations of PWH early in the pandemic. Contrary to expectations and to provider perceptions, we also found decreased tobacco and substance use and decreased suicidal ideation during the early months of the pandemic, which may reflect resiliency in this population. Care delivery is likely shifting to greater use of virtual visits over the long term, and future research should focus on longer-term outcomes and effective ways to screen for substance use and mental health symptoms in an increasingly remote-care environment.

## Data Availability

The datasets used and analyzed during the current study are available from the corresponding author on reasonable request.

## List of Abbreviations

aPR: Adjusted Prevalence Ratio
GAD-2: Generalized Anxiety Disorder
GEE: Generalized estimating equations
IDU: Injection Drug Use
MSM: Men who have sex with men
PACE: Promoting Access to Care Engagement Trial
PHQ-9: Patent Health Questionnaire
PWH: People with HIV
TAPS: Tobacco, Alcohol, Prescription medication and other Substance use instrument
